# Gravity Observations and Apparent Density Changes before the 2017 Jiuzhaigou Ms7.0 Earthquake and Their Precursory Significance

**DOI:** 10.3390/e23121687

**Published:** 2021-12-16

**Authors:** Jinling Yang, Shi Chen, Bei Zhang, Jiancang Zhuang, Linhai Wang, Hongyan Lu

**Affiliations:** 1 Institute of Geophysics, China Earthquake Administration, Beijing 100081, China; yjl@cea-igp.ac.cn (J.Y.); rular099@gmail.com (B.Z.); wlh@cea-igp.ac.cn (L.W.); lhy@cea-igp.ac.cn (H.L.); 2 Fujian Earthquake Agency, Fuzhou 350003, China; 3 Beijing Baijiatuan Earth Science National Observation and Research Station, Beijing 100095, China; 4 The Institute of Statistical Mathematics, Research Organization of Information and Systems, Tokyo 106-8569, Japan; zhuangjc@ism.ac.jp

**Keywords:** gravity change, equivalent model, apparent density change, mass transfer, earthquake precursor

## Abstract

An Ms7.0 earthquake struck Jiuzhaigou (China) on 8 August 2017. The epicenter was in the eastern margin of the Tibetan Plateau, an area covered by a dense time-varying gravity observation network. Data from seven repeated high-precision hybrid gravity surveys (2014–2017) allowed the microGal-level time-varying gravity signal to be obtained at a resolution better than 75 km using the modified Bayesian gravity adjustment method. The “equivalent source” model inversion method in spherical coordinates was adopted to obtain the near-crust apparent density variations before the earthquake. A major gravity change occurred from the southwest to the northeast of the eastern Tibetan Plateau approximately 2 years before the earthquake, and a substantial gravity gradient zone was consistent with the tectonic trend that gradually appeared within the focal area of the Jiuzhaigou earthquake during 2015–2016. Factors that might cause such regional gravitational changes (e.g., vertical crustal deformation and variations in near-surface water distributions) were studied. The results suggest that gravity effects contributed by these known factors were insufficient to produce gravity changes as big as those observed, which might be related to the process of fluid material redistribution in the crust. Regional change of the gravity field has precursory significance for high-risk earthquake areas and it could be used as a candidate precursor for annual medium-term earthquake prediction.

## 1. Introduction

High-accuracy time-variable gravity measurement is useful for understanding the changing Earth. Since the beginning of the 21st century, with the rapid improvement of gravimetric instruments and the development of dense gravity survey networks, the repeated gravity measurement has become an important approach in the monitoring of many natural phenomena, e.g., volcanic magma movement [1,2,3,4], flood surveillance [5,6], postglacial rebound [7,8], surface deformation [9], and earthquake processes [10,11,12].

Gravity observations acquired at different temporal and spatial scales have been widely used both for investigating earthquake precursor signals and for monitoring mass-transport processes. A study using GRACE satellite data revealed large-scale gravity and mass changes throughout three tectonic plates and connected slabs prior to the occurrence of the 2011 Tohoku Oki Mw9.0 earthquake [12]. In previous studies on the Tibetan Plateau, high-accuracy absolute and relative gravity measurements were used to detect anomalies [13,14,15,16,17]. Before the occurrence of the 2015 Nepal Mw7.8 earthquake, a gravitational increase of up to 22.40 ± 1.11 μGal/yr was observed at four absolute gravity stations surveyed during 2010/2011–2013, and the modeled source region of the gravity field was found be consistent with the opinion that some of the southern Tibetan Plateau crust was involved in storing the strain energy that drove the Nepal earthquake [13]. Spatiotemporal crustal density changes at different depths were observed in repeated relative gravity data acquired before the 2016 Menyuan Ms6.4 earthquake in the northeast of the Tibetan Plateau [14]. The finding indicated that the alternate state of the crustal materials near the seismic region corresponded to Amos Nur’s 1974 dilatancy–fluid diffusion model. Before the 2008 Wenchuan Mw7.9 earthquake, an increase of approximately 30 μGal was detected in repeated measurements of absolute gravity at the Pixian station, located 35 km away from the epicenter. This increase might be caused by strain and mass (fluid) transfer within a broad seismogenic source region [15]. However, comprehensive understanding of the relationship between great earthquake preparation and gravitational change remains difficult to obtain due to the complex field source factors and certain limitations in the gravity measuring systems.

Currently, the primary uncertainties of using terrestrial time-varying microgravity data for exploring crustal mass variation can be caused by gravimetric observation error, heterogenous distribution of gravity surveying networks, difficulty in separating gravitational signals, and the inherent nonuniqueness of potential field sources.

In a large-scale hybrid gravity network, many different types of relative and absolute gravimeter are used simultaneously to obtain gravity values. During gravity data processing, absolute measurement data usually acted as datum first. Accurate calibration and correction of instrumental drift are two important steps to improve the network adjustment of relative gravity observation data. Previous studies have suggested that the scale factor of a spring gravimeter can be as great as 10^−4^ per year, mainly caused by material fatigue [18], calibration shift [19], and atmospheric pressure and temperature effects [20]. It has also been reported that many spring gravimeters are subject to precision loss caused by variable drift rates in the range of 10–200 μGal [21,22]. Therefore, it is imperative that the calibration scale factor should be better constrained and the irregular nonlinear instrumental drift should be determined accurately.

Second, gravity survey stations in some regions are sparsely distributed due to difficult field conditions and transport restrictions. Moreover, gravity survey stations could be damaged by landslides or heavy rain and therefore cannot provide continuous measurements, which might affect the field source spatiotemporal resolution of the gravity observation system. Therefore, estimating the capability of the terrestrial field source monitoring system and extracting the changing field source signals from the uneven distribution of gravity measure points and earthquakes remain highly challenging tasks.

Third, gravity is an integrated parameter that includes the actions of all existing masses around the gravimetric instrument [20]. Therefore, it is difficult to separate the weak gravity signals caused by underground field changes from the gravity signal contributed by other processes, such as surface hydrological processes and vertical crustal deformation. Moreover, incomplete quantification of the contributions to gravity changes in a survey campaign limits the separation of mass redistribution effects from other geophysical processes. In short, better understanding and quantification of the effects attributable to instrumental error, the field source monitoring capability, and hydrological and atmospheric processes are prerequisite for the interpretation of mass redistribution [20].

In this study, with the data acquired in seven repeated hybrid gravity observation surveys and conducted during 2014–2017, high-precision gravity values prior to the occurrence of the Jiuzhaigou Ms7.0 earthquake (China) were obtained using the modified Bayesian gravity adjustment (MBGA) method [23]. The field source resolution of gravity network in the eastern margin of Tibetan Plateau (ETP) was quantified. The equivalent source inversion method in a spherical coordinate system was applied to obtain the variations of apparent density change of the crustal field source before the earthquake, based on the spatiotemporal joint regularization constraint model and Bayesian optimization method. An obvious north–south-trending gravity gradient zone was found within the focal area of the Jiuzhaigou Ms7.0 earthquake. Vertical crustal deformation estimated using GPS observations and near-surface water changes derived from the GLDAS model made little contribution to the gravity changes observed prior to the earthquake in the ETP. Thus, we proposed a mechanism of gravity change related to crustal material migration that agrees well with the low-velocity zone obtained from S-wave velocity structure models [24].

The remainder of this paper is structured as follows. Section 2 describes the study area, gravity data, and evaluation results derived using the MBGA method. Section 3 introduces the theory of the equivalent source inversion method. Section 4 presents the inversion results. The discussions and conclusions are provided in Section 5 and Section 6, respectively.

## 2. Data

**Figure 1:**
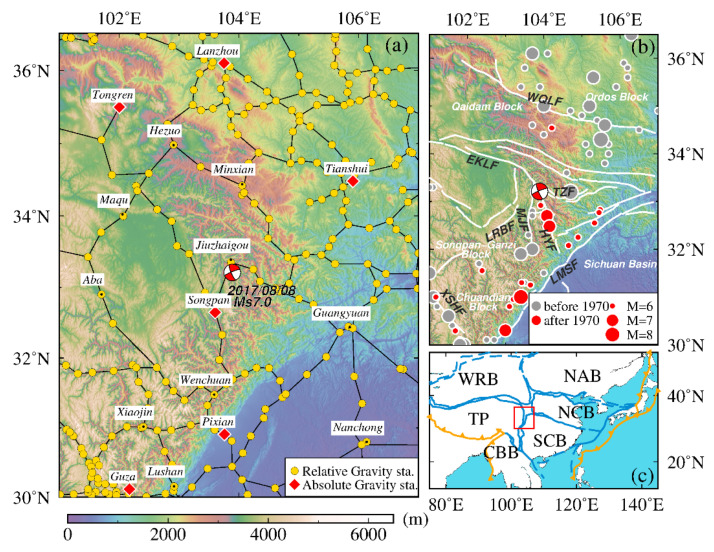
Tectonic setting and gravity survey network in the northeast margin of Tibetan Plateau. (**a**) Location of gravity survey campaign. Yellow dots indicate relative gravity observation stations, red diamonds indicate absolute gravity stations, and red-filled focal mechanism of the 2017 Jiuzhaigou Ms7.0 earthquake [25] is plotted. (**b**) Tectonic setting and historical earthquakes. Red circles represent earthquakes of ≥Ms6.0 during 1970–2020 from the China Earthquake Network Center catalog. Gray circles are historical earthquakes of ≥Ms6.0 from 780 B.C. to 1970. White lines represent major faults (WQLF: West Qinling fault; EKLF: East Kunlun fault; LRBF: Long Riba fault; MJF: Minjiang fault; LMSF: Longmen Shan fault; XSHF: Xianshuihe fault; TZF: Tazang fault; HYF: Huya fault). (**c**) Geographical position of the study area is marked by the red rectangle (WRB: West Region Block; NAB: Northeast Asia Block; NCB: North China Block; SCB: South China Block; TP: Tibetan Plateau; CBB: China–Burma Block).

The Ms7.0 Jiuzhaigou earthquake occurred on 8 August 2017 in the ETP (western China). Before this earthquake, the China Earthquake Administration had conducted a long-term gravity survey to monitor gravity changes associated with earthquakes throughout the country. The gravity survey network in the ETP is a hybrid gravity network that comprises 6 absolutive gravity stations (Lanzhou, Tongren, Tianshui, Songpan, Pixian, and Guza), 554 relative gravity stations, and 817 gravity survey segments within the region (Figure 1).

Generally, absolute gravity is measured at each absolute gravity station using an FG-5 absolute gravimeter once or twice per year. The duration of each observation is 25 h, and 100 drops are made per hour; thus, a total of 2500 repeated absolute observations are made in 25 groups. The G9 software package [26] is used to correct the effects caused by tides, polar motion, and air pressure. The accuracy of the absolute gravity measurements is approximately ±5 μGal.

The relative gravity data are acquired primarily using Scintrex CG-5, LCR-G, and Burris gravimeters. Relative gravity surveys are performed periodically, usually from March–May and from July–September, with the aim of reducing the effects of seasonal hydrological processes on the gravity values measured at each station. To reduce instrument errors, the gravity observations are conducted following a round-trip procedure (i.e., A→B→C→B→A). Generally, two gravimeters of the same type are used to perform synchronous observations at each station to improve observation accuracy. The ETP repeated gravity measurements used in this study were performed twice a year from April 2014 to April 2017.

### 2.1. Accuracy of Gravity Adjustment Results

It is believed that nonlinear instrumental drift and change of the calibration scale factor are the two most important sources that cause instrument error for spring gravimeters (e.g., CG-5, LaCoste-Romberg, and Burris). In the gravity survey campaign, the Bayesian gravity adjustment method (BGA) combined with conventional gravity adjustment (CGA) is usually adopted to resolve problems caused by the nonlinear instrumental drift rate [27]. However, in this study, the MBGA approach was applied to estimate the gravity values at each station; the scale factors and instrumental drift rates for each relative gravimeter were used in the survey campaign simultaneously [23].

In this study, to improve the adjustment accuracy and reduce the uncertainty of instrumental noise, we applied the MBGA method to estimate gravity values for all 554 stations from 2014 to 2017, using absolute gravity values as references. Details of the adjustment of the gravity dataset using the MBGA method are presented in Table 1. The accuracy of the gravity values at all stations were better than approximately 12.1 μGal. The accuracy of the gravity differences at all segments was broadly better than 14.3 μGal.

### 2.2. Drift Rates and Scale Factors Estimated Using the MBGA Method

**Figure 2:**
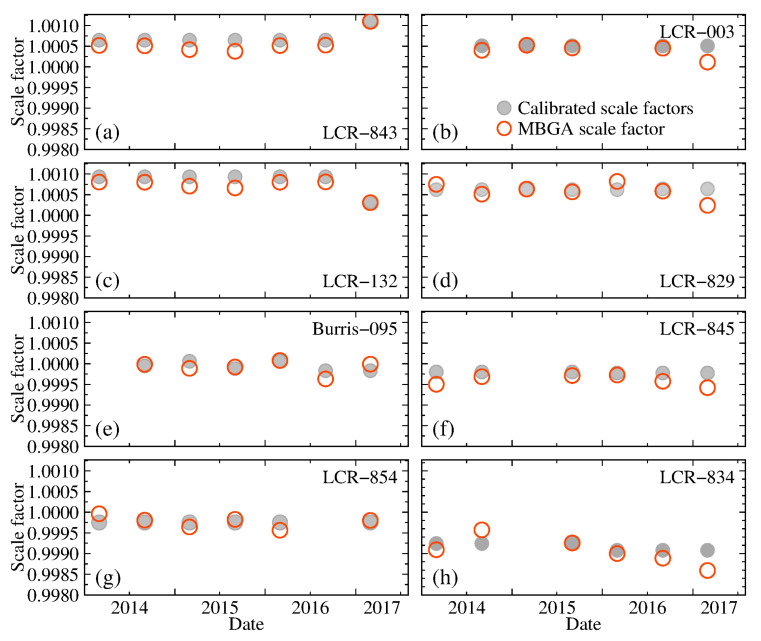
Calibrated scale factors obtained using a baseline calibration and estimated scale factors obtained based on the MBGA method. Gray circles represent the calibrated scale factors, red empty circles indicate scale factor obtained based on the MBGA method. (**a**–**h**) The deviations of the scale factors of eight relative gravimeters obtained using the two methods range from 4 × 10^−5^ to 2 × 10^−4^.

The study of microGal time-varying gravity requires a highly accurate scale factor. Calibrated scale factors obtained using a baseline calibration and estimated scale factors obtained using the MBGA method are shown in Figure 2. As shown in Figure 2a–h, the deviations of the scale factors of eight relative gravimeters obtained using the two methods range from 4 × 10^−5^ to 2 × 10^−4^. For the two relative gravimeters employed in the synchronous observations, the scale factors estimated using the MBGA method show a similar trend with the change. 

**Figure 3:**
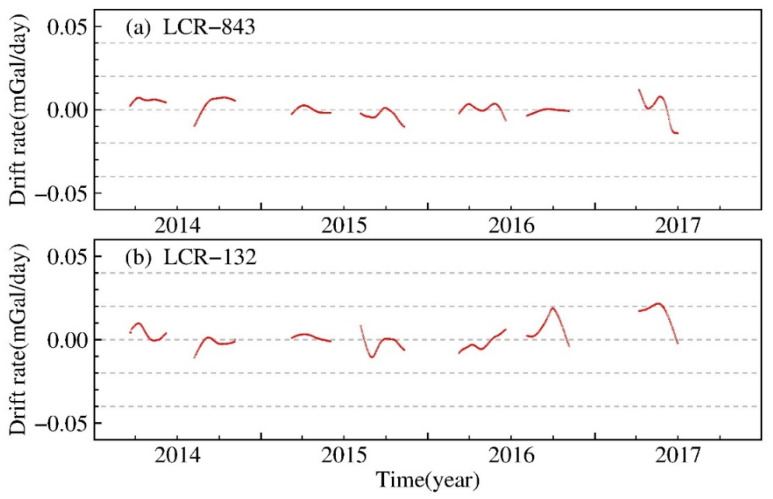
Nonlinear drift rate of LCR-843 and LCR-132 relative gravimeters obtained using the MBGA method. (**a**,**b**) The nonlinear drift rates of the two instruments, as estimated by the MBGA method, vary with an average value of approximately 0.02 mGal/d and a maximum value of approximately 0.04 mGal/d.

The gravimeter drift rate also affects the accuracy of the measured gravity values. The nonlinear instrumental drift rates of the LCR-843 and LCR-132 relative gravimeters obtained using the MBGA method are shown in Figure 3. It can be seen that both instruments were affected by irregular nonlinear drift, the rate of which increases with observation time. The nonlinear drift rates of the two instruments, as estimated by the MBGA method, vary with an average value of approximately 0.02 mGal/d and a maximum value of approximately 0.04 mGal/d, see Figure 3a,b.

**Figure 4:**
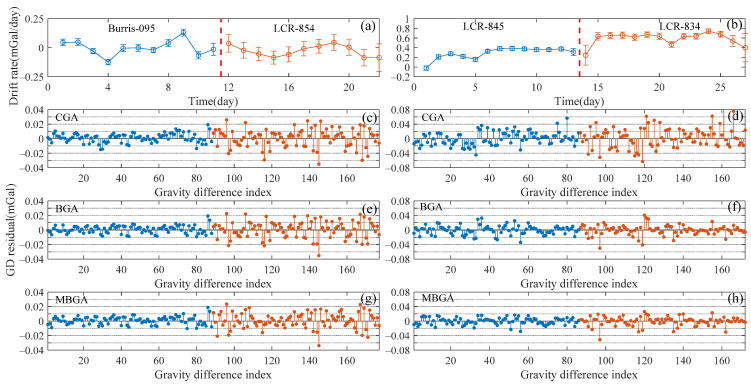
(**a**,**b**) Estimated nonlinear drift rates of four relative gravimeters. (**c**,**d**), (**e**,**f**), and (**g**,**h**) are the gravity difference (GD) residuals obtained using the CGA, BGA, and MBGA methods, respctively.

We selected four relative gravimeters operated over long periods of time and estimated their nonlinear drift rates using the MBGA method (Figure 4a,b). The estimated nonlinear drift rates of the Burris-095 and LCR-854 relative gravimeters are in the range of approximately −0.15 to 0.15 mGal/d. The drift rate of the LCR-845 instrument is approximately 0–0.4 mGal/d, while the larger drift rate of the LCR-834 instrument varies in the range of 0.2–0.8 mGal/d.

The gravity difference residuals of the instruments obtained using the CGA, BGA, and MBGA methods are illustrated in Figure 4c–h for the purpose of comparison. The gravity difference residuals of the four gravimeters are consistent with the temporal variation of the drift rates. The gravity difference residuals are proportional to the range of drift rates when applying the CGA method. The gravity difference residuals decrease from the range of 40 to 20 μGal when the BGA and MBGA methods are applied. For the LCR-845 and LCR-834 gravimeters, the gravity difference residuals obtained using the MBGA method (Figure 4h) are smaller than those derived from the BGA method (Figure 4f).

### 2.3. Cross-Validation Tests on MBGA with Absolute Gravity Data

Cross-validation analysis was performed to further verify the accuracy of the gravity values obtained using the MBGA method. In the cross-validation method, we first used certain absolute gravity values for adjustment. Then, the absolute gravity values at the other stations were compared with their corresponding estimated gravity values obtained using the MBGA method.

**Figure 5:**
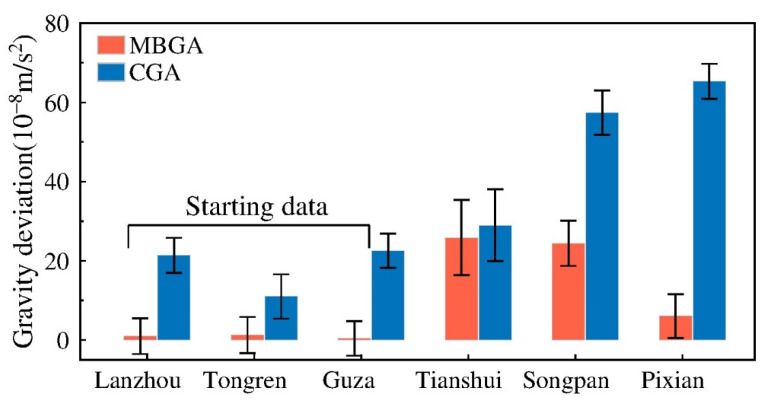
Differences in absolute gravimetry values and gravity values obtained using the CGA and MBGA method.

To evaluate the accuracy of the MBGA method, gravity data obtained in April 2015 were adjusted and evaluated using the CGA and MBGA methods. We first selected three absolute gravity datum stations (i.e., Lanzhou, Tongren, and Guza), and then gravity values at the other three gravity datum stations (i.e., Tianshui, Songpan, and Pixian) were estimated and compared with their corresponding absolute gravity values for cross-validation purposes. The differences between the estimated gravity values and the values observed by the absolute gravimeters are shown in Figure 5.

When using the CGA method, the estimated gravity values for the Lanzhou, Tongren, and Guza stations, whose gravity values are assumed to be known, deviate from the absolute gravity values by approximately 10–20 μGal. For the other three gravity stations (i.e., Tianshui, Songpan, and Pixian), whose gravity values are assumed to be unknown, the range of deviation between their estimated gravity values and the absolute gravity values is up to 60 μGal, which can be attributed to the errors of drift and scale factor.

For the MBGA method, the estimated gravity values for the Lanzhou, Tongren, and Guza stations deviate from their absolute gravity values by approximately 0–2 μGal, whereas the range of deviation for the Tianshui, Songpan, and Pixian stations is approximately 5–22 μGal. Generally, the cross-validation results indicate that the MBGA method is superior to the CGA method because of the smaller differences and errors between the estimated and observed gravity values.

The cross-validation results of all absolute gravity observations involved in the gravity survey indicate that the estimated gravity values obtained using the MBGA method deviate from the results of absolute gravimetry in the range of 0 to 20 μGal. Based on the above results, we can conclude that the MBGA method is more effective than either the BGA method or the CGA method when reducing instrumental error for hybrid gravity observations.

## 3. Methods

Gravity field inversion is a form of classical ill-posed problem. Without proper prior constraints, calculating horizontal information from inversion is meaningless. In terms of satellite gravity measurement, the equivalent water height serves as an inversed result and indicator of large-scale water storage [28]. In this study, a similar concept of the “equivalent source” was adopted to establish a relationship between the field source and the time-varying signals of the gravity field through the concept of apparent density change [29]. In the inversion equation presented in the following, smooth regularizations were introduced to constrain the spatiotemporal change of the gravity field.

### 3.1. Theory

In the equivalent source inversion method of time-varying gravity, the objective function can be expressed as follows:

(1) $$\Phi =W_{0}||Gm-\Delta g{\left(\lambda ,\theta ,z,t)|\right|}^{2}+{\Phi_{s}}^{2}\left(m\right)+{\Phi_{T}}^{2}\left(m\right),$$

where Gm−Δg(λ,θ,z,t) can be computed from the original observation dataset; the term Δg(λ,θ,z,t) is a vector comprising the observed gravity points in different survey periods, where *λ*,θ,and z are the longitude, latitude, and depth, respectively, and *t* represents the observation time; *G* is the Green function matrix for the geometric parameters of the equivalent source and the location of the gravity points, which can be calculated in advance; *m* is the apparent density vector to be inverted; *W*_0_ is a weight matrix that is inversely proportional to the noise level of the observed data; and $\Phi_{s}\left(m\right)$ and $\Phi_{T}\left(m\right)$ represent the spatial smoothness constraint and the temporal smoothness constraint, respectively, which are related to the prior assumptions of the model.

### 3.2. Regularization

(1) Spatial smoothness constraint

The spatial smoothness constraint in Equation (1) can be expanded as follows:

(2) $$\Phi_{s}\left(m\right)=W_{1}{ʃʃʃ}^{\;}\left({\left(\frac{\partial^{2}m}{\partial \lambda^{2}}\right)}^{2}+2{\left(\frac{\partial^{2}m}{\partial \lambda \partial \theta }\right)}^{2}+{\left(\frac{\partial^{2}m}{\partial \theta^{2}}\right)}^{2}\right)d\lambda d\theta dt,\;$$

where $W_{1}\;$are the weight coefficients (hyperparameters) related to the smoothness of the location and directions in space. The physical meaning is that the field source model to be solved is expected to have second-order smoothness in the λ, λθ, and θ horizontal directions, and that the smoothness degree is determined to the unknown hyperparameters $W_{1}$ that are to be optimized.

(2) Temporal smoothness constraint

The temporal smoothness constraint $\Phi_{T\;}$ in Equation (1) can be expanded as follows:

(3) $$\Phi_{T}\left(m\right)=W_{2}{ʃʃʃ}^{\;}{\left(\frac{\partial^{2}m}{\partial t^{2}}\right)}^{2}dtd\lambda d\theta ,\;$$

where $W_{2}$ is the weight coefficient (hyperparameter) related to the smoothness of time. The physical meaning is that the field source model to be solved is expected to have second-order smoothness in terms of observation time, and that the smoothness degree is determined to hyperparameter $W_{2}$.

### 3.3. Inversion

The goodness-of-fit of hyperparameters $W_{0}$, $W_{1}$, $\mathrm{and}\;W_{2}$ can be evaluated using Akaike Bayesian information criterion (ABIC) [30]. The specific ABIC calculation can be expressed as follows:

(4) ABIC=−2log(L)+2N,

where *L* is the likelihood function of the model and *N* is the number of hyperparameters. When the ABIC value of Equation (4) is a minimum, the maximum marginal probability of the m matrix in Equation (1) can be obtained together with the optimal values of the hyperparameters. In this study, we adopted the Nelder–Mead simplex optimization method [31] to solve the problem of ABIC minimization.

## 4. Results

Before applying the equivalent source inversion method to real data, its effectiveness and robustness were first assessed and verified using synthetic models at different resolutions with and without noise, on the basis of equivalent source parameters in spherical coordinates. Then, based on the optimal field source inversion parameters obtained from the model test, we obtained the crustal apparent density change results for the epicenter of the Jiuzhaigou Ms7.0 earthquake using the inversion method.

### 4.1. Checkerboard Tests

**Figure 6:**
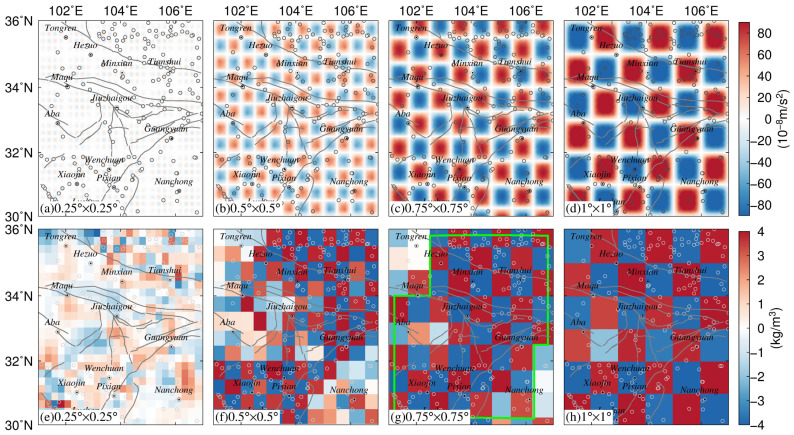
Results of checkerboard test with different spatial resolutions. (**a**–**d**) show the observable theoretical gravity anomalies with cells of different sizes (0.25°, 0.5°, 0.75°, and 1°); (**e**–**h**) show the field source densities recovered from the gravity anomalies.

This section introduces the synthetic tests performed to validate the efficiency of the abovementioned method and to evaluate the field source resolution of the gravity survey network in the ETP. The field source body was assumed to have a thickness of 1 km and depth of 15 km. Similar to the classical checkerboard test in seismic tomography, the synthetic model was generated with alternating positive and negative density perturbations of ±4 kg/m^3^. We used tesseroid cells with different sizes (i.e., 0.25°, 0.5°, 0.75°, and 1°) to discretize the source body. The observable theoretical gravity anomalies at each gravity station were calculated from the forward modeling with the assumed tesseroids (Figure 6a–d). Then, the equivalent source inversion method was applied to recover field source densities from the observable gravity changes (Figure 6e,h).

Owing to rough terrain and transport problems, the stations of the gravity survey network are not distributed uniformly. The gravity stations are mainly located in the Xianshuihe fault zone and Longmen Shan fault zone and their vicinity (Chuandian Block), whereas the distribution of stations is relatively limited in the East Kunlun fault zone and the Long Riba fault zone in the northwest of the ETP. It can be seen from Figure 6 that the inverted field source densities are highly related to both the distribution of the gravity stations and the grid size of the field source model. Obviously, Figure 6a,e show that the method cannot successfully recover the true values of the inverse parameters of the preset checkerboard model when the grid cell size is 0.25°.

It can be seen from Figure 6b,d that, in the eastern part of the study area, where the gravity stations are densely distributed, the gravity field source resolution is mainly 0.50° × 0.50°. However, there are insufficient stations to monitor the subsurface field source in western parts of the survey network, especially at near-fault stations such as Aba, Maqu, and Hezuo. Overall, the equivalent source inversion method is effective in recovering field source parameters when the gravity field source resolution is between 0.5° and 1°. According to the results of the synthetic tests at different resolutions, the optimal monitoring capability of the gravity network was determined as 0.75° in the ETP.

**Figure 7:**
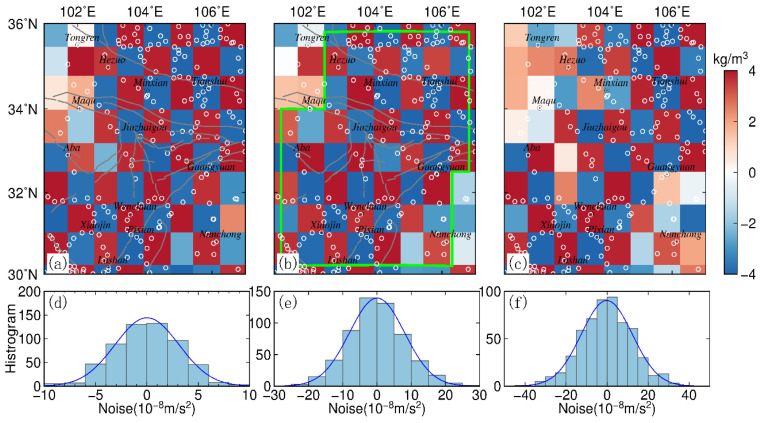
Inversed density from theoretical gravity anomalies with standard deviation of 2% (**a**), 5% (**b**), and 8% (**c**); (**d**–**f**) show histogram of the 2%, 5% and 8% Gaussian noise, respectively.

Gravity surveys in the ETP using spring gravimeters can be affected by many sources of noise, e.g., instrumental noise and environmental sources of noise. Therefore, we designed an experiment to quantify the impact of observation noise on the equivalent source inversion method. We assumed the gravity data plotted in Figure 6c were contaminated with Gaussian noise, whose standard deviation was equal to 2%, 5%, and 8% of the accurate datum magnitude (corresponding to SD = 5, 12, and 18 μGal). Figure 7 shows histograms of the noise and recovered density model derived from the noise-contaminated gravity data with a resolution of 0.75° × 0.75°.

Through comparison with the noise-free results in Figure 6c, it can be determined that the obtained inversion results shown in Figure 7 are affected by noise and that the magnitude of the effect is directly proportional to the noise level.

As shown in Figure 7a,b, the inverse field source parameters are not affected notably when the gravity data are contaminated with 2–5% noise. Especially in the Minxian, Tianshui, Jiuzhaigou, Pixian, and Lushan areas, the field source inversion results appear in good agreement with corresponding noise-free parameters shown in Figure 6c. However, when the gravity data are contaminated with 8% noise, the inversion results have less reasonable agreement with the noise-free density model in some areas with fewer stations. For example, the discrepancy is the most acute in the Aba, Maqu, and Hezuo area.

Based on the synthetic tests and the spatial distribution of the gravity survey network in the ETP, we conclude that the equivalent source inversion method is a valid algorithm that can yield reasonable results in the case of 2% and 5% noise. We outlined the area using green solid lines in Figure 6g (when the grid cell size of the tesseroid model is 0.75° × 0.75°) and Figure 7b (in the case of 5% noise), to perform the inversion more accurately.

### 4.2. Apparent Densities

**Figure 8:**
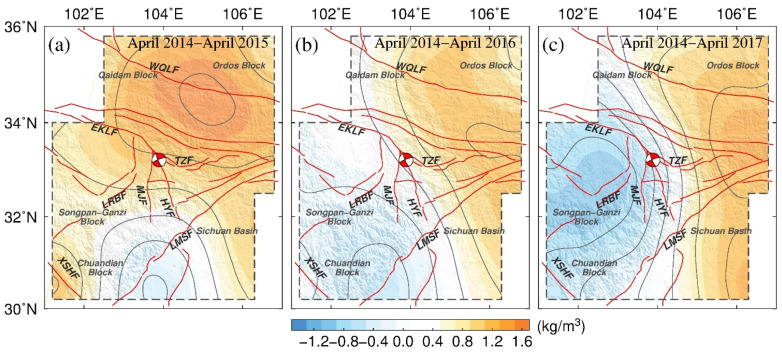
Cumulative apparent density changes during 2014–2017 before the occurrence of the Jiuzhaigou Ms7.0 earthquake (dotted lines delineate the inversion area, which is the same area as depicted by the green solid lines in Figure 6g and Figure 7b).

Using the MBGA method, we processed gravity survey data from 2014 to 2017. We used one-year time-scale data to minimize the effect of annual and semiannual signals, which could be attributable to hydrological effects and atmospheric pressure effects, amongst others. The cumulative gravity differences at each station were obtained by subtracting the gravity values of 2014/04 from those of a specific year. Then, we applied the equivalent source inversion method to the cumulative gravity differences. The inversion parameters were set as follows: the resolution of the equivalent source was 0.75° × 0.75°, the thickness was 1 km, and the depth was 15 km. The range of apparent density variations obtained by inverting the field gravity data was from −1.5 to 1.6 kg/m^3^ (Figure 8). Assuming that the average density of the crust was approximately 2700 kg/m^3^, the range of apparent density variations obtained in the inversion results was approximately 0.56–0.6‰ of the average density of the crust. The inversion area was marked by dotted lines in Figure 8, which is the same as the area depicted by green solid lines in Figure 6g and Figure 7b.

During 2014–2015, as shown in Figure 8a, positive crustal apparent densities between the Long Riba fault and the West Qinling fault could be found in the 15–16 km layer (inversion parameters) in the northern part of the study area. In contrast, negative apparent densities were observed beneath the Chuandian Block, surrounded by the Xianshuihe fault and the Longmen Shan fault in the southwestern part of the study area. Generally, the distribution of apparent densities shows that the study area was dominated by positive density change during 2014–2015.

During 2014–2016, as shown in Figure 8b, the apparent densities gradually switched from positive to negative in the region east of the epicenter, especially in the area of the Long Riba fault and the East Kunlun fault. In comparison with the values during 2014–2015, the apparent densities remained positive, but were weaker along the Ordos Block. Overall, the increase in the change to negative values approached gradually from the southwestern part of the area toward the epicenter. With consideration of the regional tectonic structure, this change could reflect migration of deep material from the west toward the east in the ETP.

During 2014–2017, as shown in Figure 8c, the negative densities continued to decrease over time and moved further toward the northwest in western parts of the study area, while the area of positive densities expanded into the Sichuan Basin. In Figure 8c, from April 2014 to April 2017, the apparent density in the western part of the survey network decreased continuously, and the area with increased apparent density expanded continuously in eastern parts. The distinct north–south-trending anomalous gradient zone was broadly centered on the Jiuzhaigou epicenter along the East Kunlun fault, Huya fault, and Longmen Shan fault zone. It can also be seen in Figure 8 that the densities changed with time in line with the eastward extension of the Tibetan Plateau.

It is important to know whether such changes still exist after the earthquakes. However, the available data currently are not sufficient to provide us with an answer to this question.

## 5. Discussion

### 5.1. Comparison of Gravity Values before and after Equivalent Source Inversion

**Figure 9:**
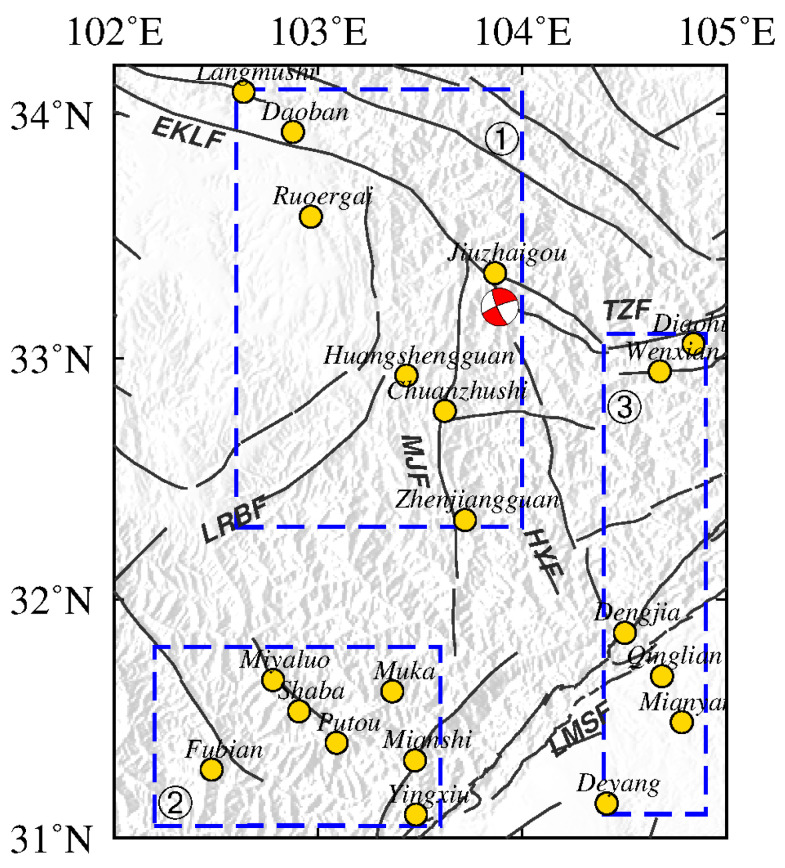
Regional distribution of gravity measuring points around the epicenter of the Jiuzhaigou Ms7.0 earthquake: ➀ northwestern region, ➁ southwestern region, and ➂ southeastern region.

**Figure 10:**
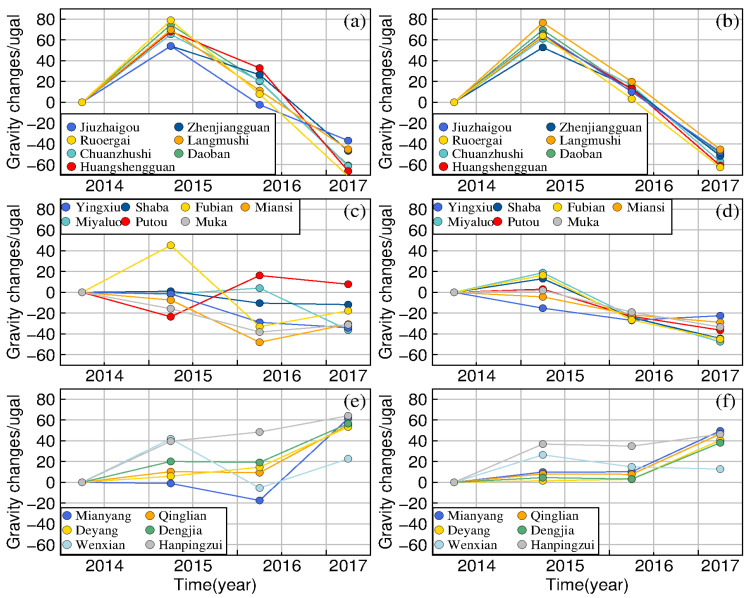
Variations of gravity values (**a**,**c**,**e**) before and (**b**,**d**,**f**) after application of the equivalent source inversion method: (**a**,**b**), (**c**,**d**), and (**e**,**f**) correspond to areas ➀, ➁, and ➂ in Figure 9, respectively.

To compare the temporal variation of forward gravity values obtained from the apparent densities with the observed gravity values, we divided the area around the epicenter into three regions (denoted as region 1 to 3), which correspond to the western region, southwest region, and southeast region, respectively, as shown in Figure 9. The temporal variation of observed gravity values and forward gravity values obtained from the apparent densities at each gravity point are presented in Figure 10. It can be seen from Figure 10 that the high-frequency components of the gravity temporal variation changes in the three regions are effectively suppressed by the spatial and temporal smoothing constraints. The inversion results appear spatiotemporally smoothed; however, the temporal variation characteristics of the gravity values in the three regions are obviously different. During 2014–2017, the gravity values observed at the seven gravity stations in region 1, such as Jiuzhaigou and Ruoergai, show synchronous trends of initial increase followed by decrease from positive to negative. In region 2, the gravity values observed at Yingxiu, Shaba, and other gravity stations (except Fubian) show a gradual trend of decrease. In region 3, the positive change rate at Mianyang, Dengjia, and the other measuring points shows an increase. As shown in Figure 10f, the high-frequency signal variations are smoother compared to the observed gravity values, which demonstrates the effectiveness of the equivalent source inversion method with spatial and temporal smoothing constraints.

### 5.2. Uncertainty Quantification

Change in the gravity field occurs not only because of movement or density changes of crustal materials, but also in response to surface deformation and the effects of near-surface hydrological processes. In this section, to establish the contribution of other field source factors in the ETP, we discuss quantitative analysis of the contribution of both vertical crustal movement using GPS observation results and local hydrological processes estimated using a global land data assimilation system (GLDAS) model [32].

#### 5.2.1. Vertical Deformation Rates

The gravity value is highly sensitive to vertical deformation [15]. Because GPS and leveling surveys can provide high-precision observations of tectonic vertical deformation, they are widely used to estimate gravity changes caused by vertical crustal movement. The GPS vertical rate field and leveling results in the ETP at different temporal scales have been observed and reported in numerous previous studies [33,34,35].

The data presented by [33,34,35] were processed following continuous and campaign GPS surveys conducted during 1996–2017. The long-term GPS vertical rates derived from these three datasets can better reflect the characteristics of the long-term vertical deformation in the ETP.

**Figure 11:**
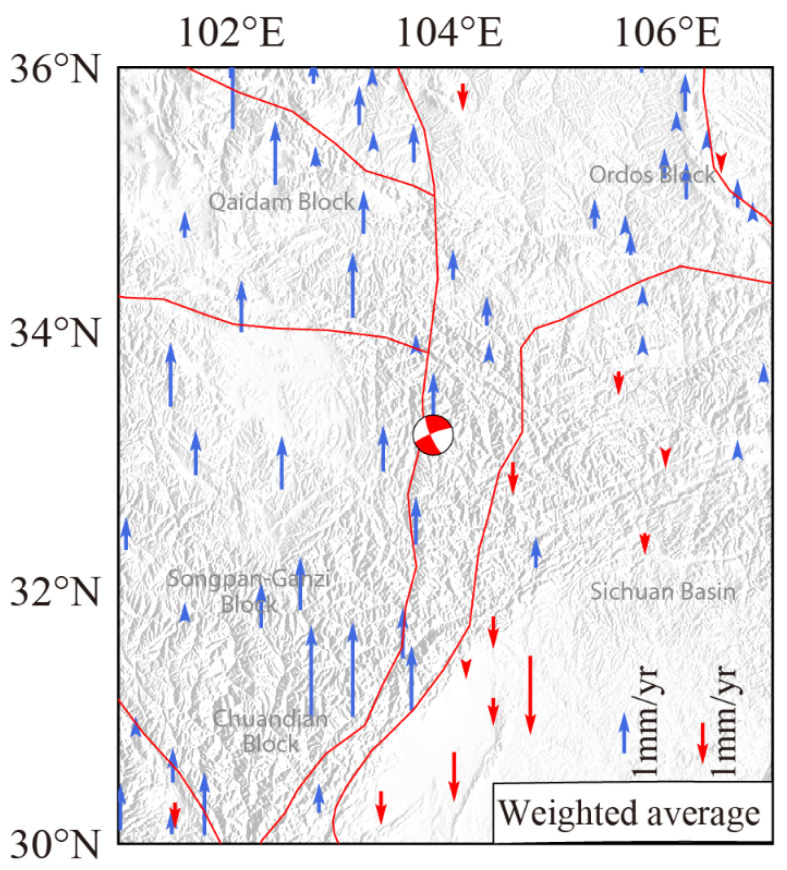
GPS vertical rates of weighted average using the datasets from [33,34,35] in the ETP. Red and blue arrows denote downward and upward GPS rates, respectively. Red lines denote the boundaries of major blocks.

To reduce the errors caused by different methods of processing GPS data, the weighted average method [36] was applied to obtain the average vertical rates of the same GPS observation stations (66 in total) from the datasets of [33,34,35]. The median errors of the observed data were taken as the weight in Figure 11.

As shown in Figure 11, there are different characteristics for the vertical motion along the block boundaries. The rate of vertical upward motion of the western Songpan Ganzi Block and the Qaidm Block is approximately 2–4 mm/yr. The average rate of subsidence of the Sichuan Basin is approximately 1 mm/yr, with a range of 0.2–2 mm/yr. The maximum crustal vertical deformation in the ETP is 4.77 mm/yr, which should account for a gravity change of approximately 1.47 μGal, assuming a value of −3.086 nm/s^2^ for the free-air gradient [37]. Moreover, leveling survey results indicate that the maximum rate of uplift in the ETP is <6 mm/yr [38]. Thus, the results from GPS and leveling surveys suggest that vertical deformation has made little contribution to the gravity change in the ETP.

#### 5.2.2. Hydrological Effects

**Figure 12:**
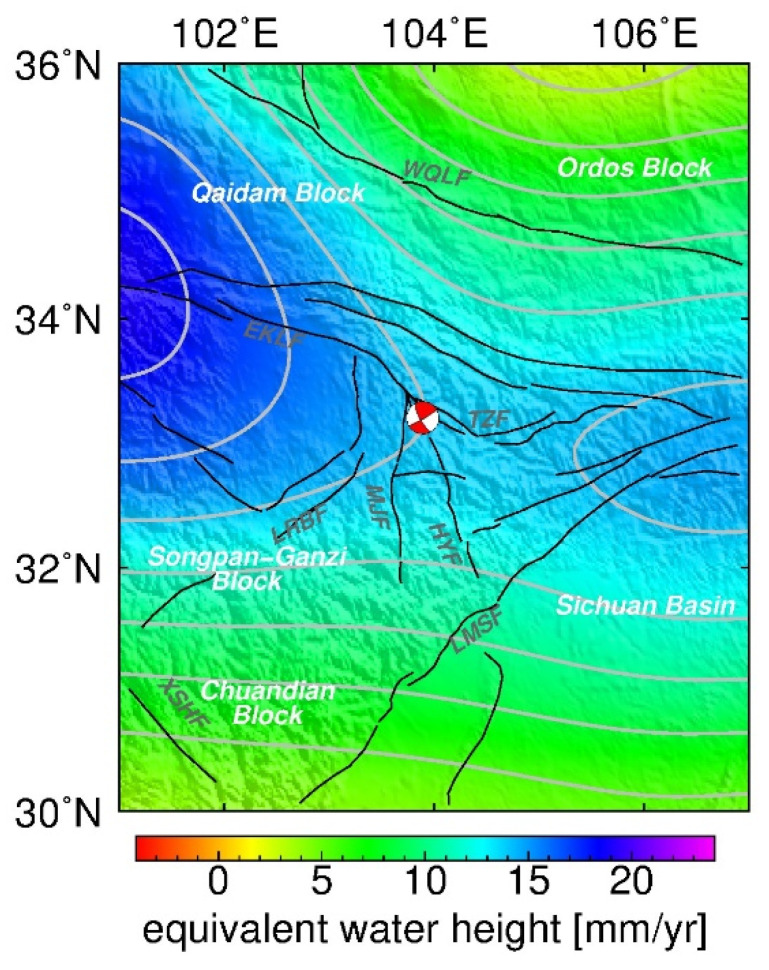
Equivalent water height rate calculated using the GLDAS model.

We also estimated the gravity changes related to terrestrial water storage change derived from the Noah land surface model in the GLDAS model during 2014–2017; the models were applied with their native 3-hourly and 0.25° × 0.25° temporal and spatial resolutions, respectively [32]. The terrestrial water storage change is the sum of precipitation, soil water infiltration, snow cover, and groundwater. It can be expressed as an equivalent height of water. Then, the Bouguer conversion ratio of 0.0421 Gal per mm of water can be used to evaluate the associated gravity change [39]. It can be seen from Figure 12 that the maximum annual equivalent water height in the ETP was in the range of approximately −3 to 19 mm/yr, corresponding to a gravity change of approximately 0.12–0.8 μGal. The GLDAS results indicate that the hydrological effect of the variation of near-surface water storage accounts for a very small proportion of the observed gravity change.

### 5.3. Implications

**Figure 13:**
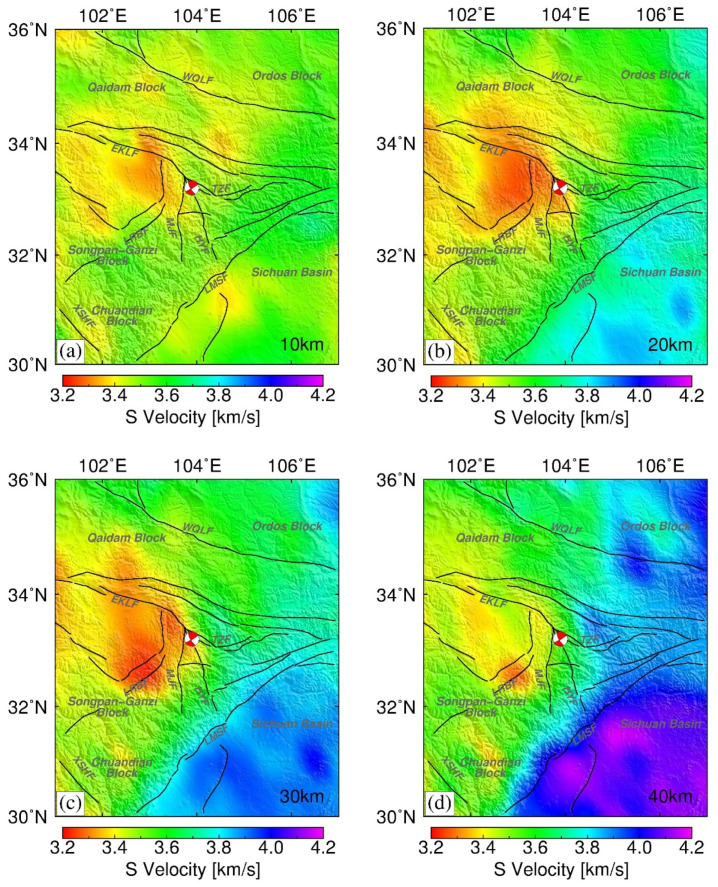
Three-dimensional shear velocity structure distribution of the ETP at four different depths: (**a**) 10 km, (**b**) 20 km, (**c**) 30 km, and (**d**) 40 km obtained from the China_2015_Vs_v1.0 model [24].

Reference seismic models with high spatial resolution of 0.5° [24] were applied to help to understand the physical mechanisms responsible for the gravity changes observed before the occurrence of the Jiuzhaigou Ms7.0 earthquake. Figure 13 shows S-wave velocity structure models at different depths of 10–40 km. At depths of 20–30 km, a large-scale high S-wave velocity anomaly is evident beneath the Sichuan Basin, whereas a prominent low-velocity zone is revealed beneath the western source region of the Jiuzhaigou earthquake.

The results of seismic tomography in this area [24] indicate the presence of low-velocity zones in the middle and lower crust in the region 1 to the west of the epicenter of the Jiuzhaigou earthquake (Figure 9). In comparison with the gravity changes in the other regions, the gravity value changes in region 1 are significant and consistent with the seismic tomography results. This might reflect the migration process of crustal materials.

At depths of 10–30 km, the distribution of S-wave velocity anomalies is similar to that of the trend of gravity change and vertical rates obtained by GPS (Figure 9). The position of the low-velocity zone coincides with the area of substantial decrease in gravity, and it is likely attributable to partial melting in the mid–lower crust.

Many researchers have investigated the crustal seismic structure of the Jiuzhaigou source area. The tomographic images exhibit a low-velocity zone (LVZ) in and around the source region in the mid-crust, characterized by a high Poisson’s ratio and a low shear wave velocity, which indicates a weak channel flow could be related to the occurrence of the Jiuzhaigou earthquake [40]. The seismogenic structure in the source region of the Jiuzhaigou Ms7.0 earthquake is characterized by high Vp/Vs ratios (high Vp and low vs. anomalies), suggesting the presence of fluids in the fractured rocks. The fluids may have contributed to the occurrence of the Jiuzhaigou earthquake by reducing the effective normal stress and the friction coefficient, thus causing the brittle failure of rocks [41].

**Figure 14:**
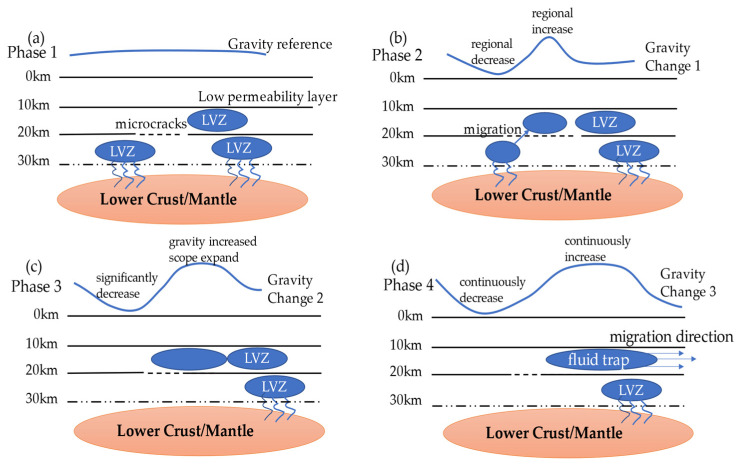
Schematic of the mechanism of cumulative gravity change and mass transfer process prior to the occurrence of the 2017 Jiuzhaigou Ms7.0 earthquake: (**a**) Phase 1 represents the gravity datum in April 2014, (**b**) Phase 2 represents the cause the of gravity change during 2014–2015, (**c**) Phase 3 represents the cause of the gravity change during 2014–2016, and (**d**) Phase 4 represents the cause of the gravity change during 2014–2017.

On the basis of the gravity change and the studies on crustal seismic structure and velocity model in and around Jiuzhaigou, a model of the gravity density variation and low-velocity zone material migration is presented in Figure 14.

In Phase 1, based on the gravity observation results in 2014, the regional crustal velocity model reveals low-velocity zones in the western focal area at the depth of 20–30 km. In Phase 2, microfractures distributed extensively in the crust provide essential channels and space for upward migration of deep fluid materials. Consequently, the observed gravity change tends to increase in eastern parts of the ETP (region 3 in Figure 8) and decrease in western parts (region 1 in Figure 8). In Phase 3, the fluid is retrapped by a low-permeability zone at the depth of 10 km; hence, the upward migration process is halted, but the fluid gradually begins to migrate horizontally. Therefore, there is a substantial decrease in gravity in the Songpan Ganzi Block (region 1 in Figure 8), and the area of gravity increase continues to expand into the Sichuan Basin (region 3 in Figure 8). In Phase 4, as the fluid continues to migrate horizontally, it merges with existing fluid at this depth to form a new low-velocity fluid zone. As the fluid moves from deep to shallow levels in this region, the gravity anomalies observed on the ground show an increasing tendency.

In conclusion, the redistribution of fluid material within the crust is considered to result in changes of the subsurface fluid environment. The fluid migration process corresponds to the formation of a substantial gravity gradient zone, which could lead to the activation of existing structures in shallower areas and induce strong earthquakes.

## 6. Conclusions

Using data from seven repeated high-precision hybrid gravity observation surveys from 2014 to 2017, gravity values before the occurrence of the Jiuzhaigou Ms7.0 earthquake were obtained with field source resolution better than 75 km based on the MBGA method. The one-year time-scale gravity values were then inverted to recover the spatiotemporal variations of crustal apparent density before the Jiuzhaigou earthquake using the equivalent inversion method, with the aim to reduce the effects of annual and semiannual signals caused by hydrological and seasonal environmental effects. With consideration of the regional vertical crustal deformation, near-surface water variation, and a regional crustal velocity structure model, we proposed a possible mechanism of crustal material migration before the occurrence of the Jiuzhaigou earthquake. The primary conclusions of this study are as follows.

(1) The differences between the estimated absolute gravity values and the observed absolute gravity values derived from a cross-validation method ranged from 0 to 20 μGal, i.e., smaller than those derived from the CGA method. Comparison revealed that the scale factor obtained using the MBGA method is more accurate than that derived from either the CGA method or the BGA method, effectively reducing the gravity difference residuals to 20 μGal. These results prove the validity of using the MBGA method to reduce the instrumental errors of scale factor and nonlinear drifts in preference to the CGA and BGA method.

(2) The effectiveness and robustness of the equivalent source inversion method was assessed on the basis of synthetic tests with different resolutions and noise levels. The results showed that the field source resolution of the gravity network in the ETP is generally better than 75 km, and that the field source parameters can be recovered effectively in the presence of Gaussian noise with a standard deviation of 12 μGal, as is usual in the gravity observation environment. We outlined the main area with a field source resolution of 75 km, which includes Hezuo and Maqu in the west of the ETP, Xiaojin in the south of the ETP, and Guangyuan and Tianshui in the east of the ETP, to perform a more accurate inversion.

(3) The range of apparent density variations obtained using the equivalent inversion method was approximately 0.59‰–0.74‰ of the average density of the crust, by setting the equivalent layer with a depth of 15 km, thickness of 1 km, and assuming the average density of the crust to be approximately 2700 kg/m^3^. The distribution of the apparent densities showed that the ETP was dominated by positive density change during 2014–2015. During the period of 2014–2016, increased negative changes approached gradually from the southwest edge of the ETP toward the epicenter of the Jiuzhaigou Ms7.0 earthquake. During 2014–2017, the negative densities continued to decrease over time and move toward the northwest in the western part of the study area, while positive densities expanded into the Sichuan Basin. The distinct north–south-trending gravity gradient zone was within the focal area of the Jiuzhaigou Ms7.0 earthquake. The densities changed with time in line with the eastward extension of the Tibetan Plateau.

(4) Prior to the occurrence of the Jiuzhaigou Ms7.0 earthquake, substantial gravity change shifted from the west to the northeast, and a distinct north–south-trending gravity gradient zone was formed within the focal area of the Jiuzhaigou earthquake. By analyzing the possible gravity changes caused by vertical crustal deformation and near surface water changes in this area, we deduced that the contributions to gravity made by these factors were too small to explain the gravity changes observed before the earthquake. The temporal gravity change and density changes suggest migration of deep material from the west toward the east in the ETP.

(5) Analysis of gravity field changes in three study areas around the epicenter of the Jiuzhaigou earthquake, using the equivalent inversion method, revealed substantial reduction in gravity change in the western focal area of the Jiuzhaigou earthquake after 2015. This decrease in gravity change was highly correlated with a low-velocity zone, which might indicate that crustal fluid migrated from the region southwest of the epicenter two years before the earthquake occurred. The gradient zone of apparent density variation coincided with the boundary area of active crust, suggesting that the variation of gravity field source is dominated by the tectonic structure of the ETP.

In summary, the MBGA network adjustment method and equivalent source inversion technique can provide an effective approach both for processing gravity data and for monitoring mass redistribution in a large-scale hybrid gravity network that includes both absolute gravity stations and relative gravity stations with resolution of 75–100 km. Regional gravity change has precursory significance for high-risk earthquake areas, and it could be used as an alternative index for annual medium-term earthquake prediction.

## Figures and Tables

**Table 1:** Results of gravity adjustment using the MBGA method in each campaign.

Survey Time	Accuracy of GV (μGal)	SD of GV (μGal)	Accuracy of GD (μGal)	SD of GD Residuals (μGal)
2014/04	8.6	3.4	6.2	7.5
2014/09	7.9	4.1	5.9	7.8
2015/04	8.5	3.2	5.7	6.7
2015/09	12.1	8.2	8.6	14.3
2016/04	9.4	12.3	6.6	8.2
2016/09	9.3	7.4	6.3	8.9
2017/04	10.8	5.1	8.4	11.2

GV: gravity value; GD: gravity difference; SD: standard deviation.

## Data Availability

Earthquake data come from the China Network Earthquake Center. The datasets generated during this study are available from the corresponding author on reasonable request.
